# Artificial intelligence deep learning for 3D IC reliability prediction

**DOI:** 10.1038/s41598-022-08179-z

**Published:** 2022-04-25

**Authors:** Po-Ning Hsu, Kai-Cheng Shie, Kuan-Peng Chen, Jing-Chen Tu, Cheng-Che Wu, Nien-Ti Tsou, Yu-Chieh Lo, Nan-Yow Chen, Yong-Fen Hsieh, Mia Wu, Chih Chen, King-Ning Tu

**Affiliations:** 1 Department of Materials Science and Engineering, National Yang Ming Chiao Tung University, Hsinchu, 30010 Taiwan, ROC; 2 Department of Materials Science and Engineering, National Chiao Tung University, Hsinchu, 30010 Taiwan, ROC; 3 National Center for High-Performance Computing, Hsinchu, 30010 Taiwan, ROC; 4 MA-Tek Inc, Hsinchu, 30010 Taiwan, ROC; 5 Department of Materials Science and Engineering, University of California, Los Angeles, CA 90095-1595 USA; 6 Department of Electrical Engineering, Tunghai University, Taichung City, 30020 Taiwan, ROC

**Keywords:** Engineering, Materials science

## Abstract

Three-dimensional integrated circuit (3D IC) technologies have been receiving much attention recently due to the near-ending of Moore’s law of minimization in 2D IC. However, the reliability of 3D IC, which is greatly influenced by voids and failure in interconnects during the fabrication processes, typically requires slow testing and relies on human’s judgement. Thus, the growing demand for 3D IC has generated considerable attention on the importance of reliability analysis and failure prediction. This research conducts 3D X-ray tomographic images combining with AI deep learning based on a convolutional neural network (CNN) for non-destructive analysis of solder interconnects. By training the AI machine using a reliable database of collected images, the AI can quickly detect and predict the interconnect operational faults of solder joints with an accuracy of up to 89.9% based on non-destructive 3D X-ray tomographic images. The important features which determine the “Good” or “Failure” condition for a reflowed microbump, such as area loss percentage at the middle cross-section, are also revealed.

## Introduction

As we enter the big data era, mobile devices are ubiquitous. Internet of things (IoT) and artificial intelligence (AI) applications are everywhere. Furthermore, in the COVID-19 pandemic period, the trend of distance learning, home office, and on-line meetings has increased the need for advanced consumer electronic products, demanding superb reliability^1–5^. With the perceived slowing down of Moore’s law of miniaturization of Si chip technology, microelectronic industry is searching alternative ways to sustain Moore’s law. 3D IC is the most promising in achieving more-than-Moore, wherein the up-scale of packaging technology is critical. There are several key interconnection technologies, such as Through-Si-via and solder bumping process. However, the fabrication defects, internal stresses, intermetallic compounds, and shear strength are still inevitable reducing the reliability of the chips^6^. Zang et al.^7^ also reviewed that the formation of intermetallic compounds (IMC), voids and cracks in many well-known materials, such as SnAg, SnBi and SnZ. These defects are also correlated with lifetime prediction for the 3D IC. Chen et al.^8^ considered certain types of solder joint geometry and generated their dataset by using finite element models. The dataset was used to train their neural networks to estimate the lifetime of the simulated solder joints and provided some design and manufacture guidelines. Thus, we ask what the technical innovations in electronic packaging for 3D IC devices will be in order to enhance performance and reliability? The answer is in AI applications to predict failure. This is because the process in 3D IC progress has been slow due to reliability testing, and the most important data collection still relies on a large amount of manual proofreading. In particular, voids cause failure in interconnects during fabrication processes and reliability tests, such as multiple reflows and electromigration^9–13^ (See Supplementary Materials).

With the rapid development in the technology of AI deep learning and the improvement of computer performance and cost, the accuracy of machine vision recognition of image targets becomes higher and higher. Convolutional neural networks (CNN) provide exceptional merits in image classification in many object recognition tasks because their network structure can extract multi-level features of images^14–18^. The promising feature of CNN is its ability to exploit spatial or temporal correlation in data. The topology of CNN is a feedforward multilayered hierarchical network that has sufficient ability to exploit spatial or temporal correlation in data^19,20^. The packaging analysis in the semiconductor industry, which used a large number of images taken by various techniques as basic data in the past, has urged to be changed. Typical non-destructive detection methods are reviewed by Su et al.^21^. Where X-ray and CT scans offer relatively high resolution of images, allowing the detection of voids, cracks and misalignment in microbumps which dominate reliability of the microbumps. To fulfil the demand for a comprehensive method for 3D IC packaging inspection and analysis, this work adopts non-destructive 3D X-ray technology to collect all the tomographic images, building a reliable database to train a dedicated AI through deep learning of convolutional neural networks. This conducts a non-destructive analysis and prediction of solder joints from an initial state to a resistance increase of about 20%. The proposed framework enables highly accurate reliability prediction via the CT images. The 3D X-ray technology can capture hundreds of out-of-the-factory microbumps in each measurement within 2 h, and our AI model can predict their status (Good or Failure) in the future, even if the reflow process has not yet been performed. This indicates that our AI model can detect the slight brightness intensity difference in the initial images of microbump and relate them to Good or Failure status after thermo tests. The proposed framework is the first and its efficiency, rapidity, and accuracy are the most advanced in the field of 3D IC reliability studies. In summary, the main contributions of the article are listed as follows:

1. The first deep learning network for predicting the status of microbump (Good or Failure) after thermal treatments based on the initial (out-of-the-factory) X-ray images.
2. Highly accurate predictions based on CT images taken by 3D X-ray technology which is non-destructive and efficient.
3. A standard procedure to assemble and calibrate sets of 3D CT images to give a 3D microbump model with a proper alignment is proposed.
4. Our analysis reveals the important features correlated with future failure of microbumps, such as the magnitude and standard deviation of brightness of intensity in the images.

## Results and discussion

**Figure 1:**
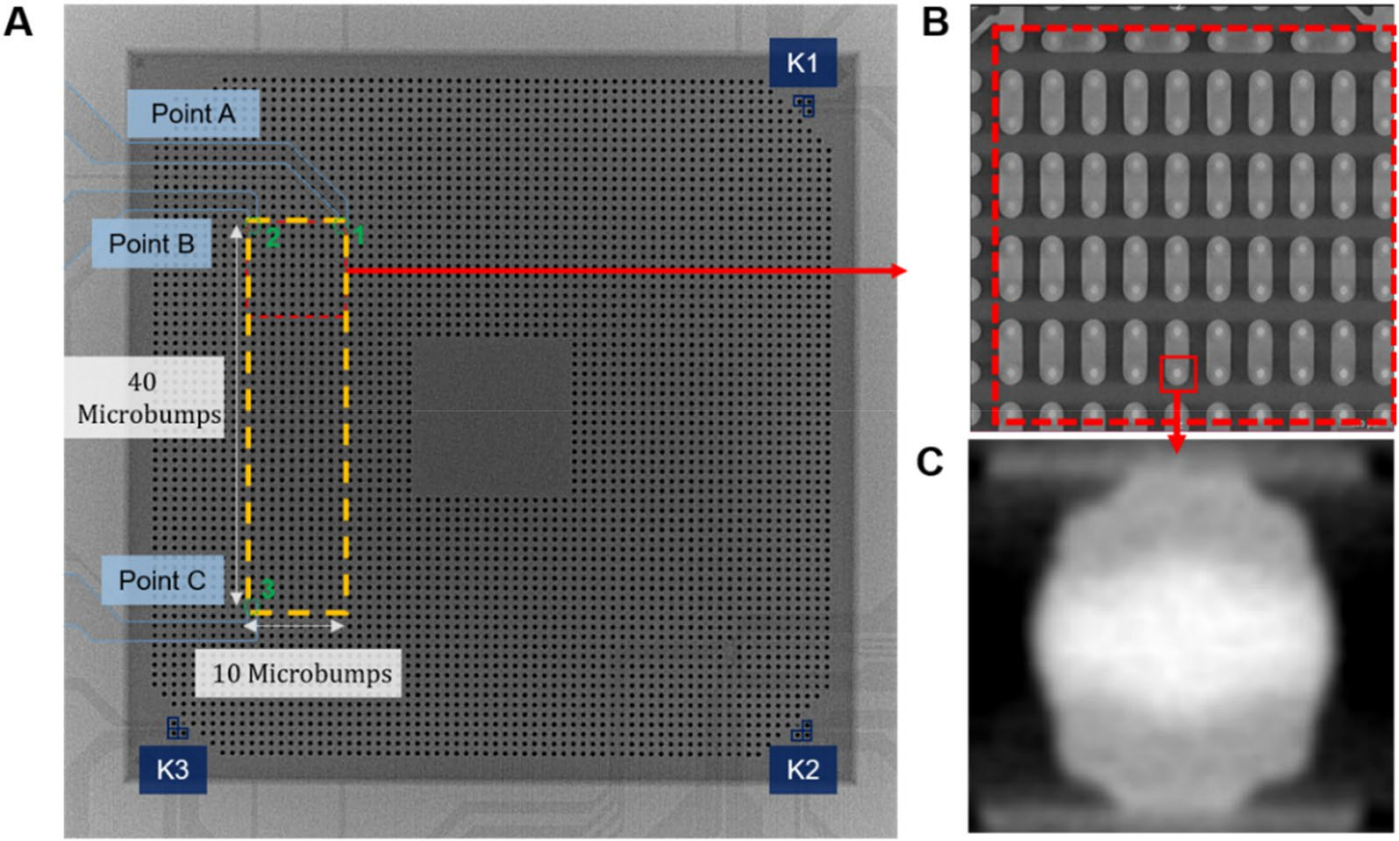
(**A**) The overall circuit diagram of a test sample by using SAT. Where the rectangular area marked by yellow dashed lines is ROI. (**B**) Zoom-in image of one-fourth of ROI containing 100 microbumps. (**C**) CT image of the cross-section of a microbump.

Figure 1A shows the overall circuit diagram of a typical test sample of 3D IC chips with solder microbumps in this work using Scanning Acoustic Tomography (SAT). The rectangular area marked by yellow dashed lines is the region of interest (ROI), including 40 × 10 pieces of microbumps. The internal structure of microbumps was scanned using 3D X-ray technology. Note that, in order to maintain the quality and the resolution of 0.85 μm of 3D computerized tomography (CT) images, each 3D CT image covered one-fourth of ROI (a red dashed rectangle in Fig. 1A) containing 10 × 10 pieces as shown in Fig. 1B. In this way, different depths of microbumps can be detected without damaging the components. The overall appearance of the wires and solder joints can also be observed simultaneously, revealing the potential areas of damage. Figure 1C shows the cross-section image of a typical microbump as an example; where the average height, and thickness of the wire, height of the under bump metallurgy (UBM), and the width of the copper trace are 61, 3, 10, and 35 μm, respectively. The overall error is within 2 μm.

After the scanning, the total resistance of all 400 microbumps in the ROI was measured through the daisy chain structure from points A to C (Fig. 1A). Where the current flows from microbumps 1 to 3, labelled in light green. In addition, the total resistance of the 40 microbumps in the left of the ROI was then measured from points B to C, where the current flows from microbumps 2 to 3. The resistance of the 400 microbumps served as the indicator of the status of the chip. According to the experimental data in the current work, the average resistance of a microbump among the 400 pieces is 15.487 mΩ.

**Figure 2:**
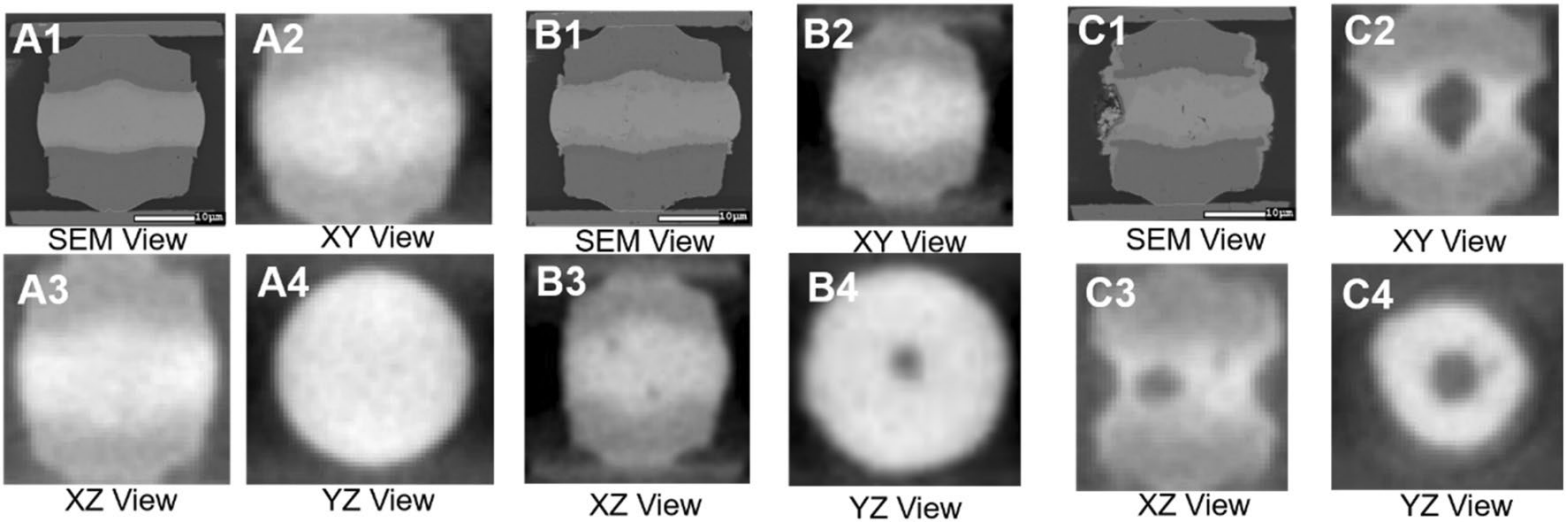
SEM images and CT images in the XY, XZ, and YZ planes of a selected microbump at (**A**) the initial state, the state of the resistance increased by (**B**) 10% and (**C**) 20%.

Next, the chip was heated in an oven, i.e. the reflow process with a heating rate at 15 °C/min until 260 °C for 30 min, and then cooled to room temperature. This heating procedure was repeated until the resistance of the 400 pieces increased by 5%. Then, the chip was scanned again to obtain 3D CT images. Thus, the internal structural changes of each microbumps in the ROI is correlated with the increase in the resistance. The scanning electron microscope (SEM) and CT images of the XY plane of selected microbumps are shown in Fig. 2. In order to illustrate the detailed evolution of the internal structure of the microbump from the initial state to the state with its resistance increased by 10% and 20%, here, the XZ and YZ planes for each selected microbump are also regenerated from its 3D solid model which is reassembled by the X-ray CT images in the XY plane. These planes are shown in Fig. 2 for illustration purposes and are not used as the training/testing dataset for the current deep learning network.

**Figure 3:**
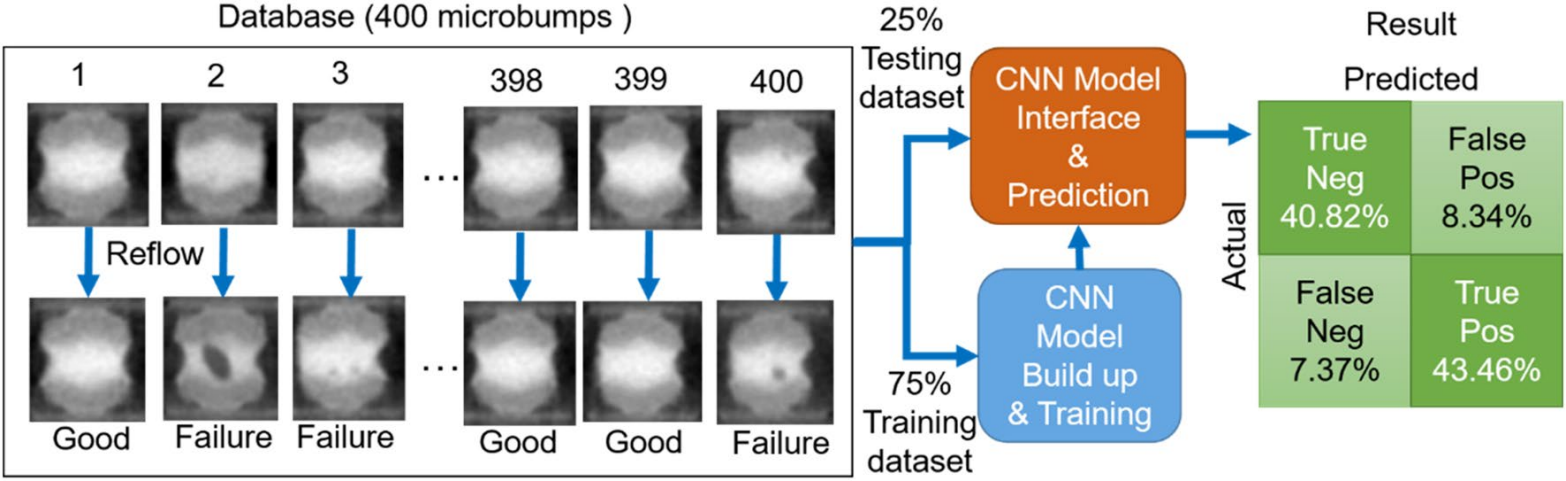
The training/testing procedures and the performance of the CNN model.

The previous experimental section generated a large number of CT scanning images showing the detail of the volumetric changes of the solder in microbumps. These images were preprocessed with six steps (See “Methods”) formulated for the raw data alignment and then served as training/testing data for our deep learning model. The training and testing procedures are summarized in Fig. 3. The entire dataset, containing 400 microbumps and their labels after the reflow process, was split randomly into 75% training and 25% testing dataset. The training dataset was then fed into the CNN for building up and training. Where architecture and parameters of the CNN were detailed in the Supplementary Materials. Next, the trained CNN model was adjusted to tackle the task for predicting the condition of the reflowed microbump based on its initial 3D image.

**Figure 4:**
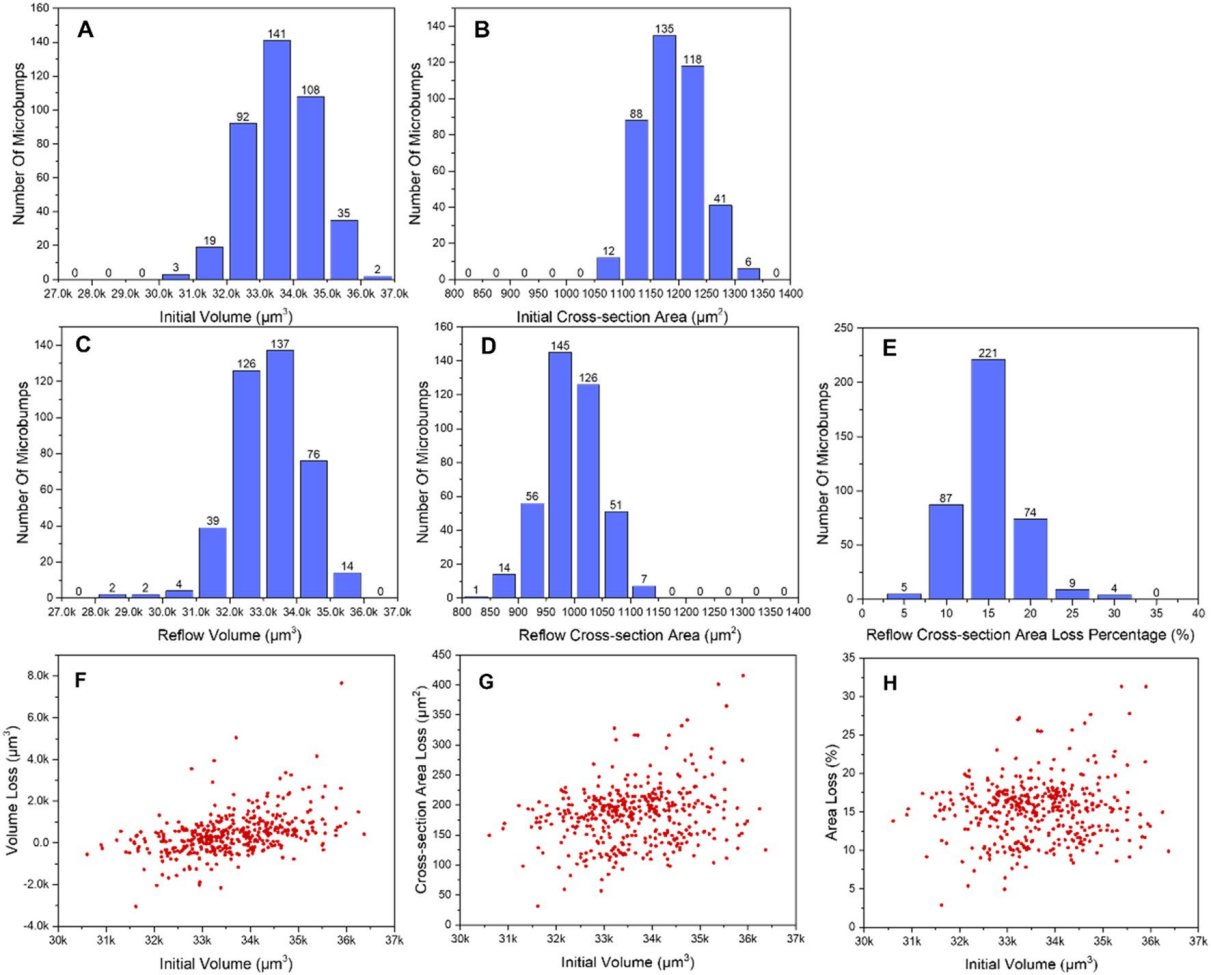
The distributions of volume and area of the cross-section of all the 400 microbumps at their (**A**), (**B**) initial and (**C**), (**D**) reflowed states. (**E**) The distribution of the cross-section area loss percentage. (**F**) The volume loss of the entire microbump, (**G**) The area loss, and (**H**) The area loss percentage at the middle cross-section versus the initial volume of all the 400 microbumps.

After the reconstruction of 3D images from the CT images of microbumps, the condition of each microbump after the reflow process should be classified and labeled. Here, three factors to reveal geometrical change between the initial and reflowed state of a microbump are chosen as the candidates measure of the classification of “Good” or “Failure.” They are volume loss of the entire microbump, area loss, and area loss percentage at the middle cross-section, where the middle cross-section is defined as the plane at the height of 30 voxels from the bottom of the 3D image with a total height of 60 voxels. Each voxel has a volume of 0.8524 × 0.8524 × 0.8524 μm^3^ according to the resolution setting in the instrument. Although the samples are printed uniformly by the instrument, there is still a slight difference between microbumps. Thus, the finished product gave a normal distribution to volume and area of the cross-section of the 400 microbumps, as shown in Fig. 4A,B, respectively. Figure 4C–E show the distribution of microbumps after the reflow process in terms of the volume, cross-section area, and cross-section area loss percentage, respectively. By comparing Fig. 4A,C, it can be observed that the reflow process only causes a slight shift between the two distributions, i.e. an insignificant volume change of the two states. In contrast, Fig. 4B,D display a remarkable shift between the two states. Thus, in respect of overall distribution, area change of the middle cross-section reflects more significantly on the effect due to the reflow process than volume change.

We next examine the relationship between the three factors and the initial volume. Figure 4F shows that the volume loss range is mostly between − 2000 and 2000 μm^3^ (except for some extreme cases). This indicates that the resulting volume changes due to the reflow process are, in fact, very close to the typical error (around 3%) that occurred in the experiments when measuring the 3D volume by X-ray. This can also be seen that some values of the volume loss are negative, i.e. volume increases after the reflow process, and they are also within around 3%. In addition, the values of volume change of microbumps in Fig. 4F mainly accumulate around zero regardless of their initial volume, indicating a poor correlation between the two. In contrast, data points in Fig. 4G,H do not accumulate around zero, showing greater variation throughout the minimum to the maximum initial volume of the microbumps. Also, the values of area loss and area loss percentage of the cross-section are all positive values, i.e. area of cross-section of microbumps decreases after the reflow process, and this is in reasonable agreement with the experiments.

Based on the results reported above, the two factors (area loss and area loss percentage at the middle cross-section) were used to determine the condition/label of reflowed microbumps. It is worth noting that the CNN model trained by area loss percentage has better accuracy than that trained by area loss value. Thus, in the current work, area loss percentage is chosen to be the primary factor determining the “Good” or “Failure” condition for a reflowed microbump. Here we set that whenever the area loss percentage between the initial and reflowed state was less than 16% (referring to Fig. 4E), the microbump was labeled as “Good”, while it greater or equal to 16%, the microbump was labeled as “Failure.”

The performance of the current CNN model on the testing data is shown on the right-hand side of Fig. 3. The True Negative and True Positive are our target regions, showing that the true ratio of the prediction reaches more than 80%. The results indicate that the proposed CNN model can predict and accurately classify the condition of a given microbump after the reflow process JUST based on its initial (before the reflow process) 3D image.

Moreover, our network design enables the interpretation of the current predictions. We use the Deep Taylor Decomposition (DTD)^29^ method to find out what our model had learned in the training process. The DTD method decomposes the output of a deep neural network in terms of their input variables based on the Taylor expansions. Two examples are illustrated in Figs. S9 and S10, showing the cross-sectional images and the corresponding DTD values for a “Good” microbump and a “Failure” microbump, respectively. Voxels with higher DTD values mean they have a higher weighting in our model, i.e. the predictions of “Good” or “Failure” for a reflowed microbump are dominated by these voxels. The results show that the materials near the boundary and the boundary itself of the microbumps are the most important parts for its future status after reflow process.

After applying the DTD method, the voxels with the same DTD value are selected and then we cropped a 15 × 15 × 15 bounding box around the selected voxels to analyze their neighbouring voxels. Then, the intensity of voxels in each bounding box is calculated and the average intensity density and the standard deviation of the intensity distribution for all the bounding boxes are determined, as shown in Fig. S11. The results show that the intensity distribution for the “Failure” microbumps have a greater standard deviation than that for the “Good” microbumps when their DTD value greater than 0.25. This indicates that microbumps with non-uniform and rough material distribution around the boundary are more likely classified as “Failure” after reflow process. It also makes sense as these roughness may generate higher resistance. This also indicates that our model can detect the subtle brightness intensity difference in the initial images of microbump and correlate them with the status after thermo tests.

It is also worth noting that the proposed approach is extremely efficient. With the optimized scanning parameters and fixed image resolution of 0.85 μm, the 3D X-ray technology used in the current work is able to scan the area containing 10 × 10 microbumps at a time within about 2 h, which indicates that all the microbumps are taken in single CT scan. Here, we consider 400 microbumps in two different stages (initial and reflowed). Thus, it requires about 16 h to complete the scanning for the entire dataset used in the current work, i.e. 2.4 min for a microbump in average. After the images were taken, we segmented every micro-bump by applying image processing method and further analysis for each of them. In addition, the CNN model can predict results in seconds. Finally, the proposed approach can be considered extremely efficient, rapid in practice for the field of 3D IC reliability studies.

In summary, we conducted a non-destructive failure prediction of solder joints in electronic packaging technology, used computerized tomography to obtain a large amount of image data, built a reliable database based on these data, and trained a dedicated analytical AI model through deep learning. The relevant image values can be adjusted so that they can meet the sampling standards of the AI model. With the improvement of image verification, the accuracy of AI predictor after 3D CNN can also be effectively improved. The results showed that the initial recognition and prediction capabilities of the AI model trained through this technology have reached 89.9%. With the massive expansion of image data, it is expected that the accuracy will be further improved. This approach has greatly shortened the experimental time in the reliability prediction of microbumps.

## Methods

### Experiment procedures

Solders have been critical materials as vertical interconnects in 3D IC integration^2,20^. As the miniaturization of the microelectronic devices, the dimension of the solder joints also continues to scale down. The diameter of a typical flip-chip solder joint is approximately 100 μm, as shown in Fig. S1A, and it has been reduced to 20 μm for microbumps in 3D IC integration, which has been adopted for high performance devices, such as high computing devices and artificial intelligent (AI) chips. Figure 1B shows the schematic drawing for the dimension for a microbump adopted in this study. Its diameter is 30 μm with a solder height of 12 μm. Figure 1C presents the cross-sectional SEM image for a microbump. The most striking feature for the microbump is the significant reduction in solder volume, which is approximately only 1/100 of that in the flip-chip solder joint.

Several process and reliability may arise due to the small amount of solders in a microbump, including voiding and necking induced by side wetting of solder on Cu and Ni metallization^10,22–24^, solder extrusion during bonding process^25^, voiding due to electromigration^9,13,26^, and porous Cu_3_Sn formation^27^. Figure S2 shows a microbump after solid state aging at 150 °C for 473 h. Some of the solders diffused to the side wall of the Cu/Ni metallization and caused necking in the solders. As the dimension of the microbump scales down, the amount of solder decreases. The necking will become worse and cause resistance to increase, even open failure in the solder joints^10,28^.

The test vehicle consists of a top Si die bonded to a bottom Si die, with 4548 microbumps between the two chips. To bond the microbumps, the two chips were aligned and reflowed at 260 °C for 1 min. Figure S3A illustrates the layout of the test vehicle, with 6 mm × 6 mm for the top die, and 15 mm × 15 mm for the bottom die. The pitch for the microbumps is 80 μm. Figure S3B shows the plan-view X-ray image for the dashed rectangle area in Fig. S3A. Arrays of microbumps can be clearly seen. For reliability tests, the chip was heated in an oven at a heating rate of 15 °C/min, held for various time durations, and then cooled down to room temperature 25 °C. After the reflow processes, the resistance of the daisy chains was measured. The resistance increase was between 5 and 20% of its initial value in this study.

Non-destructive observation on the microbumps was conducted by 3D X-ray. The model is ZEISS Xradia 620 Versa with 0.8 μm spatial resolution. The 3D images can be analyzed from various directions and cross-sections. Figure S4A shows the computerized tomography (CT) images along the XZ plane for 10 neighboring as-fabricated microbumps. There was no necking or voiding in the solders. After reflow at 260 °C for 60 min, the resistance of the daisy chains increased 16.4% of its initial value. Then the same specimen was observed by the 3D X-ray again. The microstructures of the microbumps were illustrated in Fig. S4B. Serious necking occurred in all the 10 microbumps, indicating the 3D X-ray is able to observe the morphological changes of the microbumps.

### Data preparation and training strategy

The architecture of 3D convolution neural network (CNN) employed in this work is illustrated in Fig S5 with detailed parameters listed in Table S1. The CNN structure involves an input layer, hidden layers, and an output layer. In the hidden layers, the input data of 3D CT images are abstracted in a convolutional layer (a learnable filter) with the ReLU activation function to polled feature maps followed by the max-pooling. After passing through four learnable filters, the predicted outputs are produced by the fully connection layer. To optimize the performance of the current CNN model for the predicted accuracy, the raw input data have to be in line with following six steps. First, the CT images of each microbump were assembled and cropped (where copper wires were neglected) to form a 3D image with 60 × 60 × 60 voxels using Avizo. 400 pairs of 3D image for the initial and reflowed state of a microbump were generated. Second, the distribution of brightness intensity of the two 3D images for the initial and reflowed state of any given microbump should be aligned and matched, i.e. image histogram matching, to enable a better learning efficiency of the deep learning model. Thus, the intensity distribution of the reflowed image was scaled to match the three crucial points of the distribution: the minimum (threshold), most proposed intensity, and brightest points. Third, the positions of the two 3D images for the initial and reflowed state of any given microbump should also be aligned and matched. This was achieved by rotating the reflowed image to align the moment of inertia and the corresponding axes of the two 3D images. This procedure can minimize the shifting and mismatch of the images between the two states. Fourth, all the reflowed microbumps were labeled based on the factor corresponding to the geometrical loss. Whenever the factor was less than a preset threshold, the microbump was labeled as “Good”, while it was greater than the threshold, the microbump was labeled as “Failure”. Fifth, the amount of the labeled data was further enlarged by data augmentation. Where the 3D images were rotated every 10° of *θ* and *φ* in Euler angles and cropped to maintain the original size, generating more than 20,000 data points, which are sufficient for the training of the current neural network.

## Supplementary Information

Supplementary Information.

## Data Availability

All data generated or analyzed during this study are included in the published article and Supplementary Information, and are available from the corresponding authors upon reasonable request.

## Code availability

All code was implemented in Python using Tensorflow as the primary deep learning package. All codes and scripts to reproduce the experiments of this paper are available from the corresponding authors.
