# Supplementary material for: Artificial intelligence deep learning for 3D IC reliability prediction
Source: Sci Rep. 2022 Apr 25;12:6711. doi: 10.1038/s41598-022-08179-z (PMC9035975; doi:10.1038/s41598-022-08179-z)

**Artificial intelligence deep learning for 3D IC reliability prediction**

Po-Ning Hsu^1,2†^, Kai-Cheng Shie^1,2†^, Kuan-Peng Chen^3^, Jing-Chen Tu^6^, Cheng-Che Wu^1,2^, Nien-Ti Tsou^1,2*^, Yu-Chieh Lo^1,2^, Nan-Yow Chen^3*^, Yong-Fen Hsieh^4^, Mia Wu^4^, Chih Chen^1,2^, and King-Ning Tu^5*^

^1^Department of Materials Science and Engineering, National Yang Ming Chiao Tung University, Hsinchu, Taiwan 30010, ROC.

^2^Department of Materials Science and Engineering, National Chiao Tung University, Hsinchu, Taiwan 30010, ROC.

^3^National Center for High-performance Computing, Hsinchu, Taiwan 30010, ROC.

^4^MA-tek Inc., Hsinchu, Taiwan 30010, ROC.

^5^Department of Materials Science and Engineering, University of California, Los Angeles, CA 90095-1595, USA.

^6^Department of Electrical Engineering, Tunghai University, Taichung City, Taiwan 30020, ROC

^*^Corresponding author. Email: kntu@ucla.edu (K.T.); nanyow@nchc.narl.org.tw (N.C.); tsounienti@nctu.edu.tw (N.T.)

^†^These authors contributed equally to this work.

**Supplementary information**


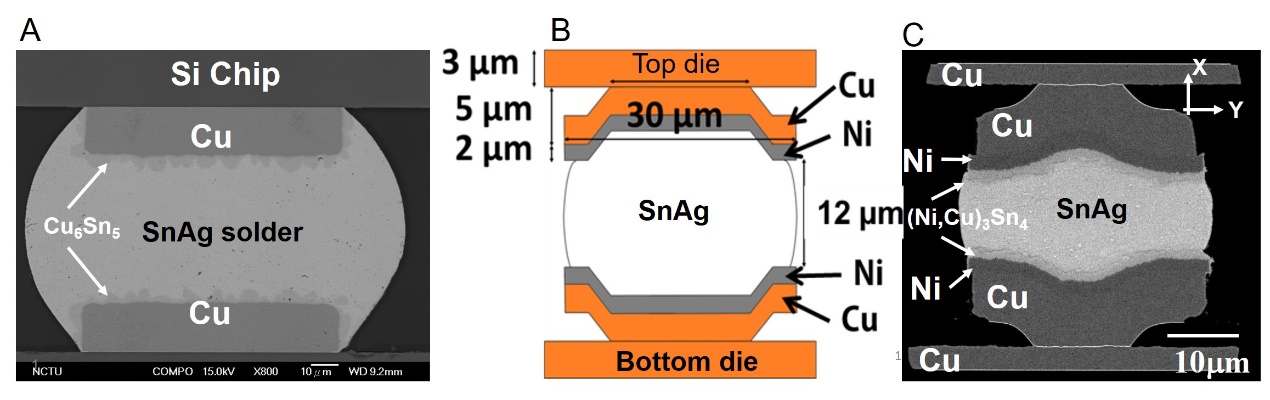


**Figure S1. Dimension of a flip-chip solder joint and a microbump.** (A) Cross-sectional scanning electron microscope (SEM) image for a flip solder joint. (B) Schematic drawing showing the dimension of a microbump. (C) Cross-sectional SEM image for a microbump.


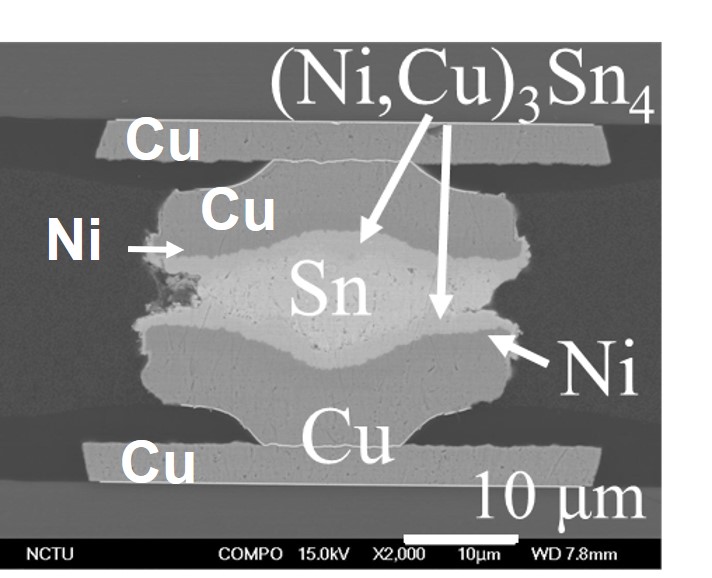


**Extended Data Figure S2. Necking in a microbump due to side wall wetting of solder on Ni and Cu metallization**. The microbump was annealed at 150 °C for 473 h.


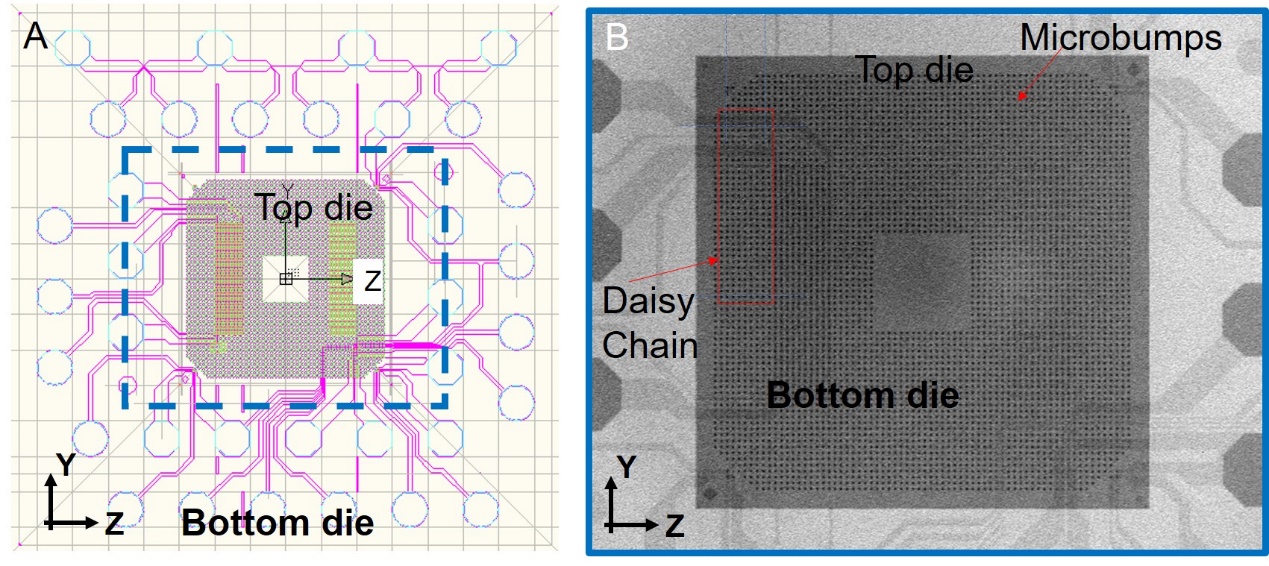


**Extended Data Figure S3. Layout of the test vehicle.** (A) Layout for the top and bottom Si dies. (B) 2D X-ray image for the microbumps. The daisy chains with 400 microbumps located at the upper-left side of the vehicle.


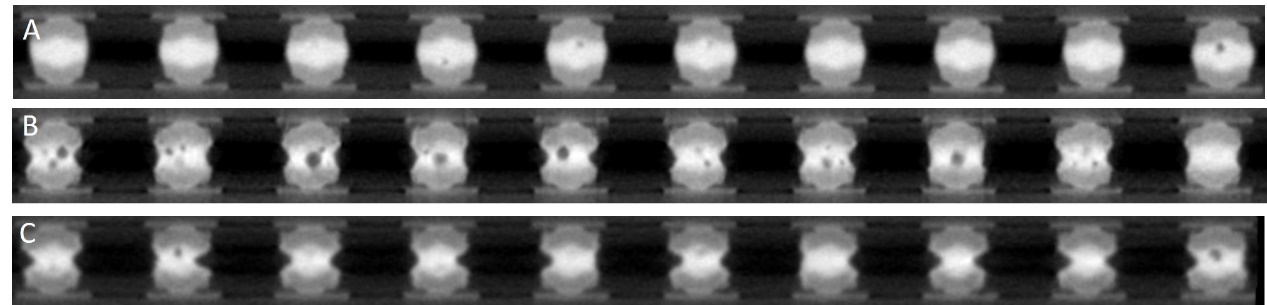


**Extended Data Figure S4**. **Non-destructive observation by 3D X-ray on the microbumps before and after reliability tests.** (A). Ten as-fabricated microbumps. (B). The microbumps in Fig. 4A after the reflow at 260°C for 30 min. (C). The microbumps in Fig. 4B after additional reflow at 260°C for 30 min.


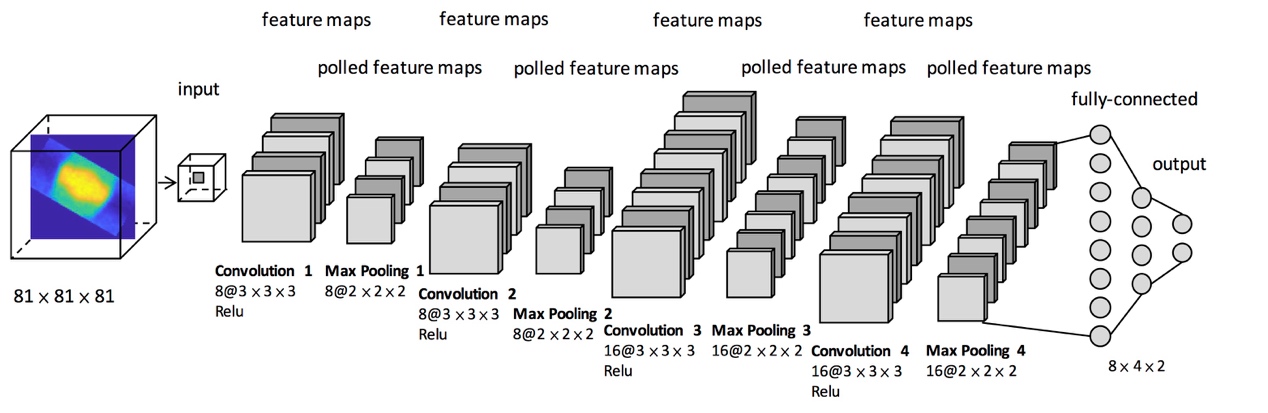


**Extended Data Figure S5. Structure of CNN model in the current work.**


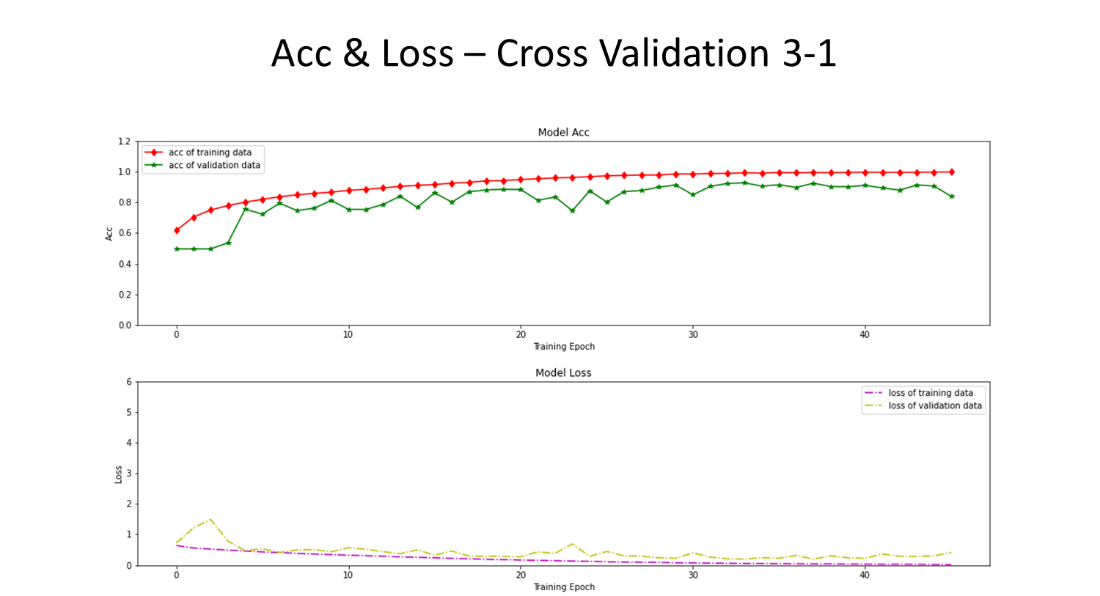


**Extended Data Figure S6. The accuracy and loss vs. number of epoch in cross validation 1 among three.**


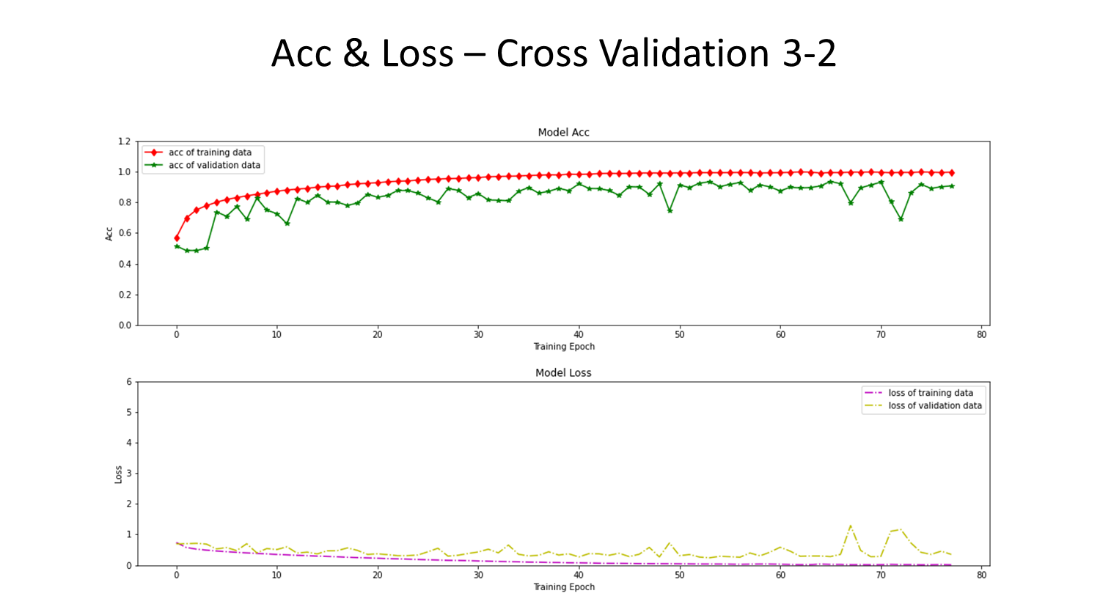


**Extended Data Figure S7. The accuracy and loss vs. number of epoch in cross validation 2 among three.**


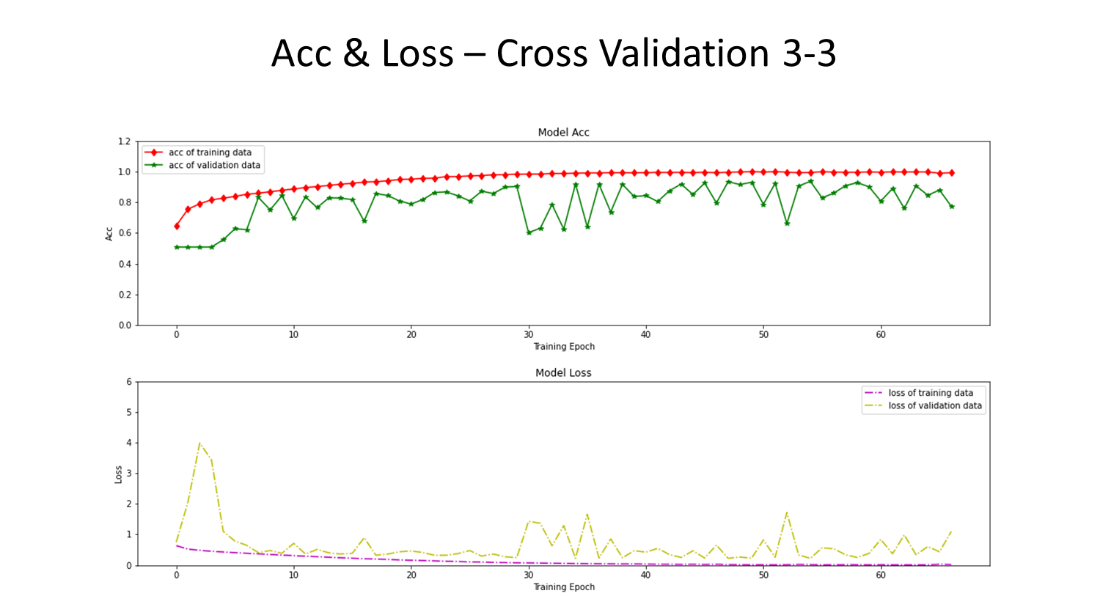


**Extended Data Figure S8. The accuracy and loss vs. number of epoch in cross validation 3 among three.**


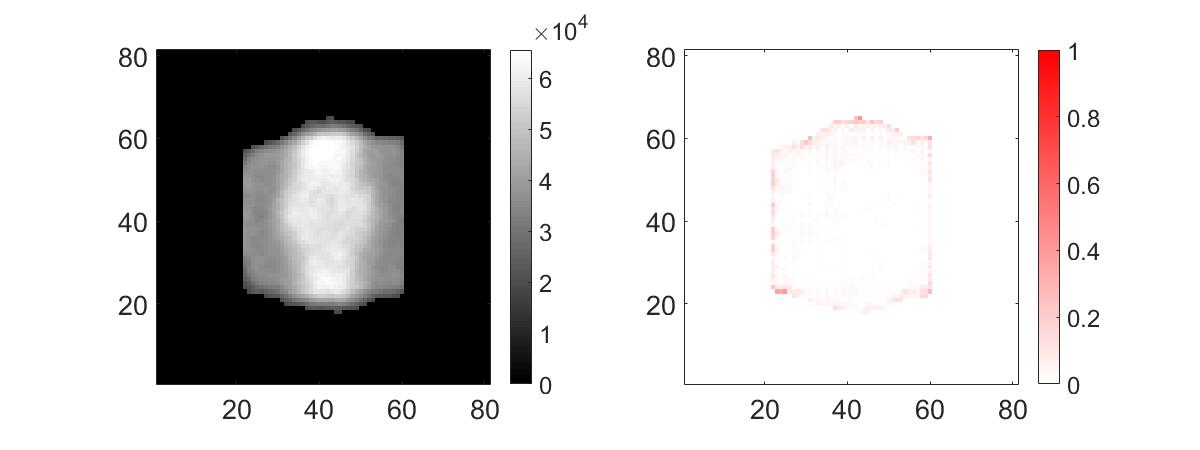


**Extended Data Figure S9.** The cross section image and its corresponding DTD values for a “Good” microbump.


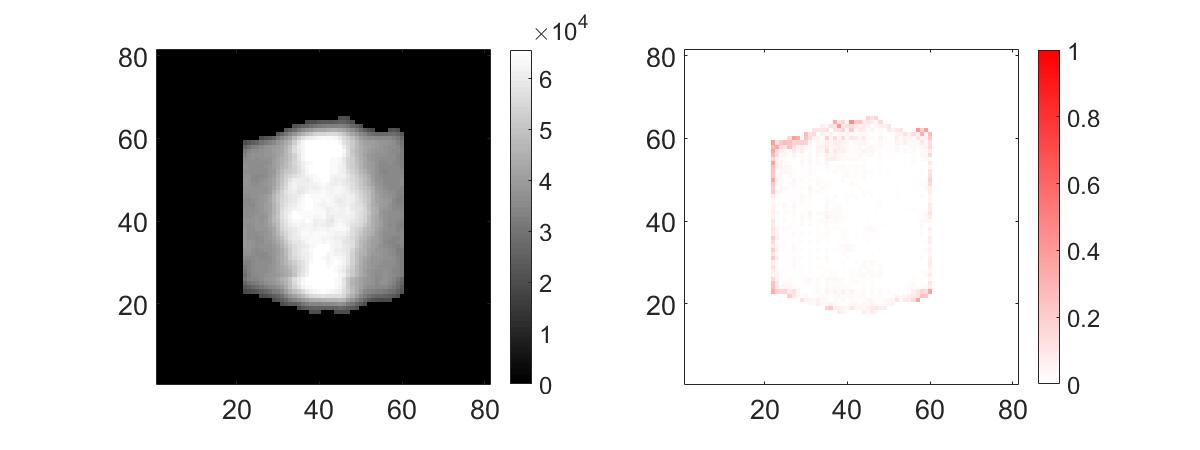
 **Extended Data Figure S10.** The cross section image and its corresponding DTD values for a “Failure” microbump.


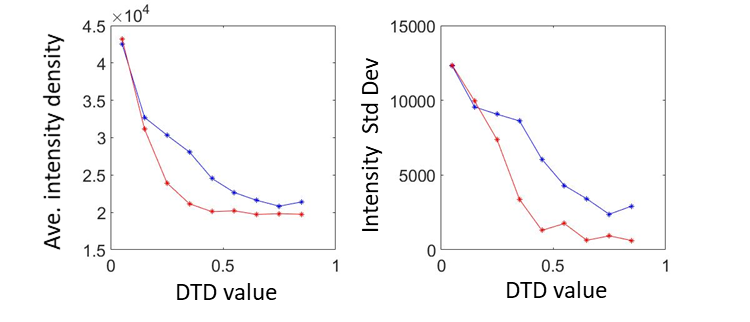


**Extended Data Figure S11.** The average intensity density and the standard deviation of its intensity distribution for different DTD values. The red and blue lines are the results of “Good” and “Failure” microbumps, respectively.

**Extended Data Table S1. Parameters of CNN model in the current work.**


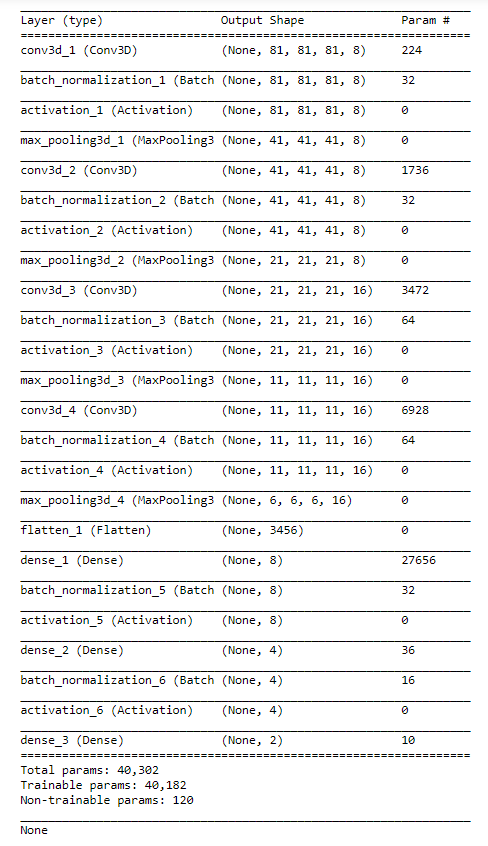

Supplement: Supplementary file 1 — Supplementary Information. [file 41598_2022_8179_MOESM1_ESM.docx]
